# Circadian Blood Pressure Variations Computed From 1.7 Million Measurements in an Acute Hospital Setting

**DOI:** 10.1093/ajh/hpz130

**Published:** 2019-08-16

**Authors:** Adam Mahdi, Peter Watkinson, Richard J McManus, Lionel Tarassenko

**Affiliations:** 1 Institute of Biomedical Engineering, Department of Engineering Science, University of Oxford, Oxford, UK; 2 Nuffield Department of Clinical Neurosciences, Oxford University Hospitals NHS Trust, Oxford, UK; 3 Sensyne Health, Schrödinger Building, Heatley Road, Oxford Science Park, Oxford, UK; 4 Nuffield Department of Primary Care Health Sciences, University of Oxford, Oxford, UK

**Keywords:** blood pressure, circadian rhythms, hospital measurements, hypertension

## Abstract

**BACKGROUND:**

Knowledge of the circadian blood pressure (BP) variations in the acute hospital setting is very limited.

**METHODS:**

This is a retrospective analysis of BP data for in-hospital patients stratified by age and sex. We used data collected with the help of a standardized electronic health record system between March 2014 and April 2018 on the adult general wards in 4 acute hospitals in Oxford, UK.

**RESULTS:**

A total of 41,455 unique patient admissions with 1.7 million sets of vital-sign measurements have been included in the study. The typical 24-hour systolic BP profile (dipping pattern during sleep followed by a gradual increase during the day) was only seen in the younger age groups (up to 40–49 for men and 30–39 for women). For older age groups, there was a late nocturnal rise in systolic BP, the amplitude of which increased with age. The late nocturnal BP rise above the age of 50 was seen whether or not the patient was treated for or previously identified with hypertension.

**CONCLUSION:**

Hospitalized patients’ circadian patterns of BP largely mirror those found in the community. High-quality hospital data may allow for the identification of patients at significant cardiovascular risk through either opportunistic screening or systematic screening.

## INTRODUCTION

### Background

It is well known that in the community blood pressure (BP) is characterized by fluctuations occurring over different time scales.^1^ Rather than representing random phenomena, those changes are the result of interactions between environmental and behavioral factors and the cardiovascular regulation system.^2^ These give rise to long-term (clinic or visit-to-visit) variability,^3^ mid-term (day-to-day) variability, usually assessed by home monitoring,^3^ or short-term variability, known as circadian BP variation and assessed by 24-hour ambulatory monitoring.^4^ These fluctuations have prognostic importance. Recent analysis^5^ of data from a registry-based, multicenter, national cohort that included 63,910 adults recruited over a decade showed that measurements made over 24 hours with ambulatory BP monitoring (ABPM) were a stronger predictor of all-cause and cardiovascular mortality than clinic BP measurements.

Nighttime BP profiles can be classified into 3 patterns: dippers (subjects in whom BP falls at night), nondippers (subjects in whom BP does not fall at night), and risers or reverse dippers (subjects in whom BP rises at night).^6,7^ These categories have been observed from studies using ABPM,^8,9^ home BP monitoring,^10,11^ and a small-sample study in elderly hospitalized patients.^12^ The riser or reverse dipping pattern has been found to be more common in older patients.^13–15^ A systematic review by Taylor *et al*.^4^ found nighttime dipping was associated with a lower risk of cardiovascular events while rising BP at night compared with daytime values was associated with increased risk.

Measurements taken while in hospital have historically been recorded on paper charts. The advent of electronic vital-sign recording allows patterns of BP recorded in hospital to be analyzed at scale. The presence of multiple readings when patients are hospitalized may allow more reliable estimation of 24-hour BP profiles. Also, the stress associated with being in hospital may mimic laboratory studies suggesting that increased BP lability under stress itself predicts future cardiovascular events.^16^ Finally, being in hospital may present an opportunity to assess BP control while receiving prescribed medication.

### Objectives

In this article, we determine the 24-hour BP profiles in the hospitalized adult population stratified by age and sex using a large database of 1.7 million sets of observations. We assess whether those 24-hour BP profiles are affected by the presence of a diagnosis and treatment for hypertension or affected by observations taken when patients are physiologically unstable.

## METHODS

### Study design

Our study is a retrospective analysis of BP data for in-hospital patients stratified by age and sex. Linked prescription data were available from the electronic patient record.

### Participants

**Figure 1.**
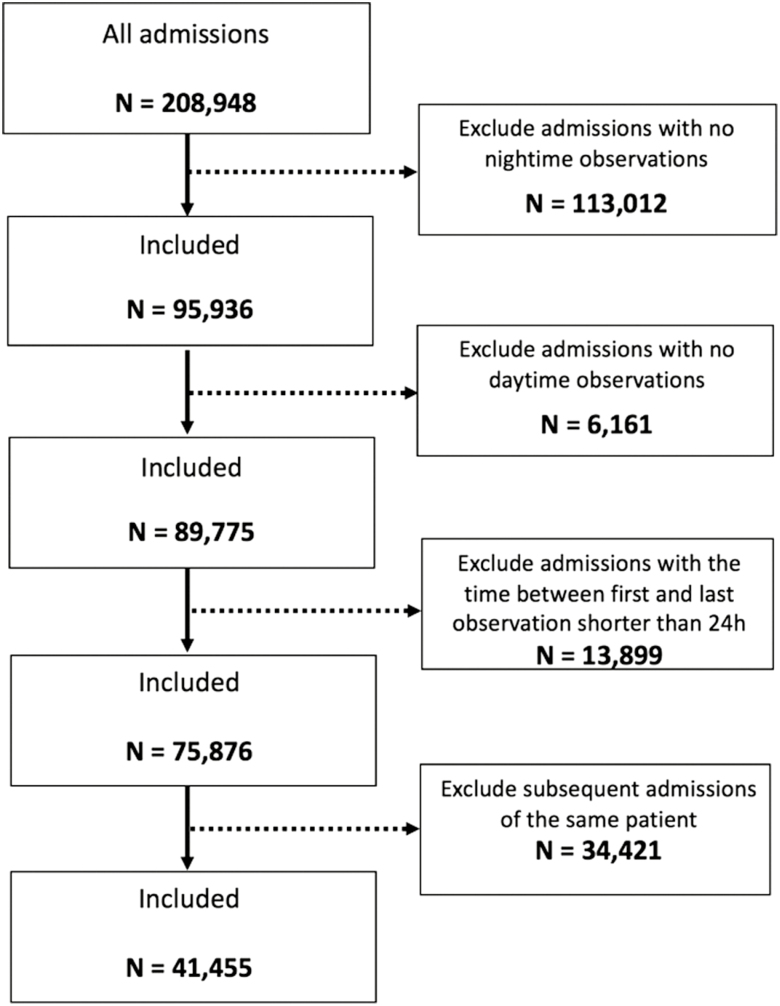
Inclusion/exclusion criteria flowchart.

All patients aged 16 years and older admitted between March 2014 and April 2018 were eligible for the study, which was approved by the Oxfordshire Research Ethics Committee (reference: 16/SC/0264), with Confidential Advisory Group approval to process patient data without consent (reference: 16/CAG/0066). We included BP measured on all the adult wards (excluding the intensive care units) in 4 acute hospitals in the Oxford University Hospitals (OUH) NHS Foundation Trust. We included the first admission of each patient that had at least 3 observations, with at least one during the nighttime (defined as from midnight to 5:59 am) and at least one during the daytime (defined as from 10:00 am to 7:59 pm), with 2 observations at least 24 hours apart (Figure 1). The nighttime and daytime periods are defined in accordance with standard practice in the literature.^7^

### Data sources

Patients had their vital-sign data, including heart rate, systolic and diastolic BP, collected using a standardized electronic health record system, System for Electronic Notification and Documentation (SEND), which records vital-sign values, calculates an Early Warning Score^17^ and enables the recognition of physiological deterioration on the ward.^18^ In the calculation of the Early Warning Score, each vital sign was assigned a weight from 0 to 3 depending on the degree of abnormality.

Nurses and health care assistants within OUH NHS Trust measure patients’ vital signs and receive regular training updates. The patient position in which BP is taken is optional. OUH NHS Trust policy recommends that observations should be undertaken a minimum of 12 hourly, with the observation frequency increasing to 4 hourly and then hourly depending on the degree of abnormality as defined by the Early Warning Score. All vital-sign equipment used within the study was purchased and maintained (including regular calibration) in accordance with the Oxford University Hospital Trust Medical Devices Management Policy. Other data (date of birth, sex, ICD-10 codes, and prescription information) were obtained from the Patient Administration System (PAS) in the hospitals’ electronic patient record, Cerner Millennium (North Kansas City, MO; https://www.cerner.com/).

### Data analysis

Each participant’s BP and heart rate data were averaged by hour of the day. Individuals therefore contributed only averaged data for a maximum of 24 one-hour intervals regardless of their length of stay in hospital and number of measurements recorded. Supplementary Figures S1 and S2 illustrate typical systolic BP recordings taken during hospital stay. For the patient of Supplementary Figure S2, for example, 4 systolic BP measurements were taken between 6:00 am and 6:59 am (and plotted at hour 6:00 am in the figure) during the entire hospital stay. Subsequently, these were averaged (red line) and hourly values of averaged systolic BP constructed in a 24-hour plot (with some empty fields, the shaded regions in Supplementary Figures S1 and S2, for the 1-hour intervals for which no BP was recorded).

Mean population BP and heart rate stratified by age and sex were plotted for each hour of the day, providing summary 24-hour profiles by decade of age. To assess whether vital signs taken when patients were physiologically unstable affected our findings, we repeated this analysis by removing all observation sets, which contained a vital sign other than BP having a weight greater than 0.

We also investigated the effects of antihypertensive medication or a diagnosis of hypertension on the 24-hour BP profile. We again aggregated all the averaged data by hour of the day for each patient within 2 separate groups: (i) normotensive elderly group—patients above the age of 60 who did not have a prescription for any antihypertensive drug as listed in the British National Formulary^19^ (Supplementary Table S4) and who did not have an admission ICD-10 code for hypertension (Supplementary Table S5), (ii) hypertensive elderly group—patients above the age of 60 on at least one antihypertensive drug, and/or with an admission ICD-10 code for hypertension. We then compared the 24-hour BP profiles of the 2 groups, separated by sex. This analysis was restricted to patients over 60 years of age, as the hypertensive group was predominantly found within this age bracket. Finally, we explored whether differences in 24-hour profile exist between elective and emergency admissions.

The differences in BP between 2 time points were assessed using 2-sample *t*-test for difference of means.

## RESULTS

The overall dataset included 41,455 admissions of unique patients with a total of 1,701,812 BP sets of vital-sign measurements (Figure 1).

### Descriptive data

Table 1 shows patient demographics. The median length of time between the first and last BP measurement was 4.7 days, over which time a median of 27 BP measurements was taken. The frequency of admission by hour of the day is shown in Supplementary Figure S6. A more detailed description, including different age groups and hypertension categories, is also available (Supplementary Table S1).

**Table 1.** Demographic descriptors

Variable	All
Number, *N* (%)	41,455 (100)
Sex, men, *N* (%)	20,169 (49)
Age [years], mean (SD)	64 (19)
LOM [days], median (IQR)	4.7 (7.4)
Observations, median (IQR)	27 (34)
Risk factor	
Charlson Comorbidity Index, median (IQR)	3 (10)
In-hospital mortality, *N* (%)	2,233 (5)
Theater admissions, *N* (%)	20,480 (49)
Admission method	
Emergency, *N* (%)	26,290 (63)
Elective, *N* (%)	13,490 (33)
Other, *N* (%)	1,675 (4)
Specialty	
Medical, *N* (%)	18,113 (44)
Surgical, *N* (%)	22,543 (54)
Other, *N* (%)	792 (2)
Hypertension category	
Normotensives, *N* (%)	19,312 (47)
Hypertensives, *N* (%)	22,143 (53)

LOM (length of measurements) denotes the time between the first and last blood pressure measurements taken during the hospital stay. IQR, interquartile range.

Patients 50–89 years of age accounted for nearly three-quarters of all admissions (70.1%). The number of men and women included in the study was similar (49% men and 51% women) with a median age of 63 years for men and 64 for women.

The frequency of BP measurements and the average value of systolic and diastolic BP, across the entire cohort, calculated for each 1-hour interval are shown in Table 2. The fewest measurements were taken between 3:00 am and 3:59 am (13,578), with the greatest number of measurements taken between 5:00 am and 5:59 am (34,158) before nursing hand-over and again soon after the nightshift starts, between 8:00 pm and 8.59 pm (32,467). The ratio between the maximum and the minimum numbers of measurements per hour was 2.5:1.

**Table 2.** Frequency of measurements, systolic blood pressure values, and diastolic blood pressure values

Hour	0	1	2	3	4	5	6	7	8	9	10	11
Frequency	17,663	17,364	15,524	13,578	22,488	34,158	29,171	20,332	21,260	27,509	29,961	25,414
Mean SBP	125	125	125	125	128	130	130	130	127	125	125	125
SD SBP	23	23	23	23	21	20	20	22	21	19	18	19
Mean DBP	67	67	67	67	69	70	70	70	69	68	68	69
SD DBP	13	13	14	14	12	11	11	13	13	11	11	12

Hour	12	13	14	15	16	17	18	19	20	21	22	23
Frequency	22,558	23,702	26,832	30,151	29,598	27,069	24,829	29,012	32,467	28,211	22,311	19,127
Mean SBP	125	124	125	126	128	128	128	128	128	128	127	126
SD SBP	20	20	19	19	19	20	21	19	19	20	21	23
Mean DBP	68	67	68	69	69	69	69	69	69	69	68	67
SD DBP	12	12	11	11	11	12	12	11	11	11	12	13

SBP, systolic blood pressure (mm Hg); DBP, diastolic blood pressure (mm Hg).

### Twenty-four-hour BP variation in hypertension and normotension

**Figure 2.**
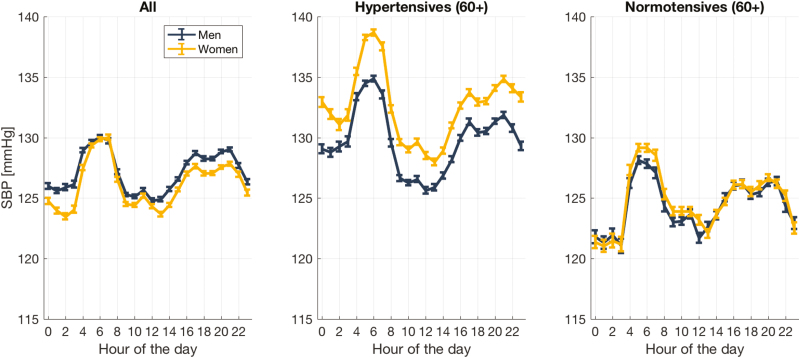
Twenty-four-hour (mean ± SE) systolic blood pressure profile for all admissible cohort (left); hypertensives above the age of 60 (center); and normotensives above the age of 60 for men and women.

The 24-hour systolic BP variations for the overall hospitalized population (20,169 men and 21,286 women) is shown in Figure 2 (left). The general cohort experienced a late nocturnal rise in mean systolic BP between 2:00 am and 5:59 am for both men (3.8 mm Hg (95% confidence interval, 0.5)) and women (5.9 mm Hg (95% confidence interval, 0.6)) with *P* < 0.001 (Table 3).

**Table 3.** Mean systolic blood pressure (mm Hg) change between 2:00 am and 5:59 am for different cohorts of patients

	Men			Women		
Cohorts	SBP_2:00_ (95% CI)	SBP_5:00_ (95% CI)	Difference (95% CI)	SBP_2:00_ (95% CI)	SBP_5:00_ (95% CI)	Difference (95% CI)
All	125.9 (0.3)	129.7 (0.3)	3.8 (0.5)	123.5 (0.3)	129.4 (0.3)	5.9 (0.6)
Hypertensives (60+)	129.3 (0.5)	134.5 (0.4)	5.2 (0.9)	131.1 (0.5)	138.3 (0.4)	7.2 (0.9)
Normotensives (60+)	122.0 (0.7)	128.1 (0.6)	6.1 (1.1)	121.5 (0.7)	129.2 (0.5)	7.7 (1.1)
20–39 years	122.3 (0.7)	121.0 (0.6)	−1.3 (1.3)*	111.3 (0.6)	112.2 (0.5)	0.9 (1.2)**
40–59 years	124.2 (0.6)	126.0 (0.5)	1.8 (1.1)	117.9 (0.6)	121.5 (0.5)	3.0 (1.1)
60+ years	127.3 (0.4)	132.7 (0.3)	5.4 (0.7)	128.1 (0.4)	135.4 (0.3)	7.3 (0.7)

SBP_2:00_, average systolic blood pressure (mm Hg) measured between 2:00 and 2:59 am; SBP_5:00_, average systolic blood pressure measured (mm Hg) between 5:00 and 5:59 am (mm Hg); difference, difference between mean systolic blood pressure (mm Hg), SBP_2:00_, and SBP_5:00_; CI, confidence intervals.

All differences are significant with *P* < 0.001 except *P < 0.15 and **P < 0.05.

The 24-hour systolic BP variations for hypertensive (9,102 men and 9,208 women) and normotensive (3,688 men and 4,328 women) patients of 60 years and older are shown in Figure 2 (center, right). Both hypertensive and normotensive cohorts experienced similar late nocturnal rises (between 2:00 am and 5:59 am) in mean systolic BP for both men and women (Table 3).

### Twenty-four-hour BP variation for age categories

**Figure 3.**
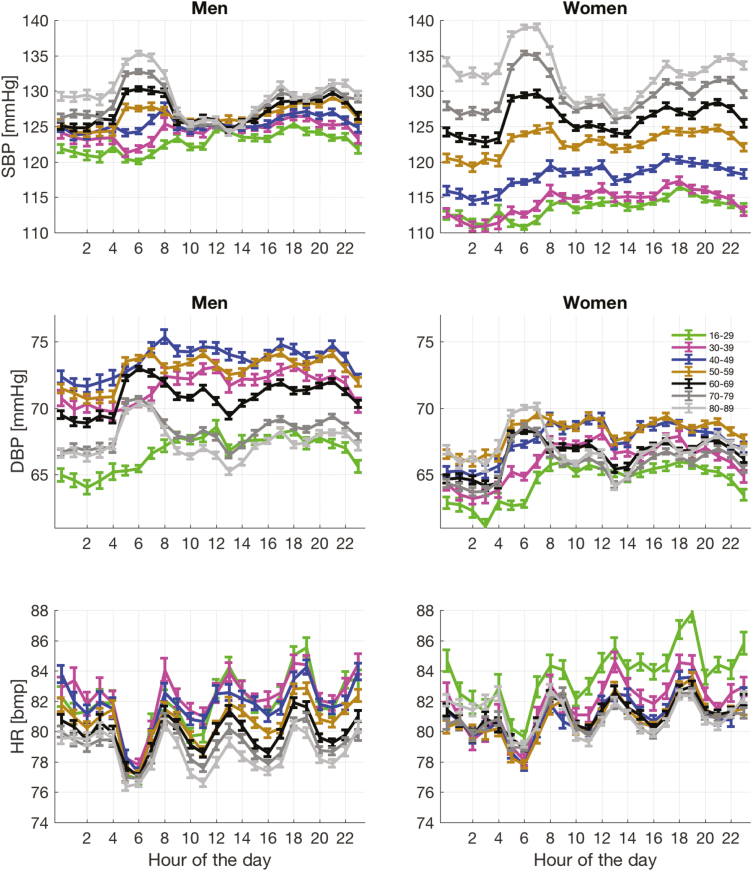
Twenty-four-hour (mean ± SE) systolic (top row) and diastolic (middle row) blood pressure profiles and twenty-four-hour (mean ± SE) heart rate variations stratified by age (18–29, 30–39, 40–49, 50–59, 60–69, 70–79, 80–89 and 90+) for men and women.

**Figure 4.**
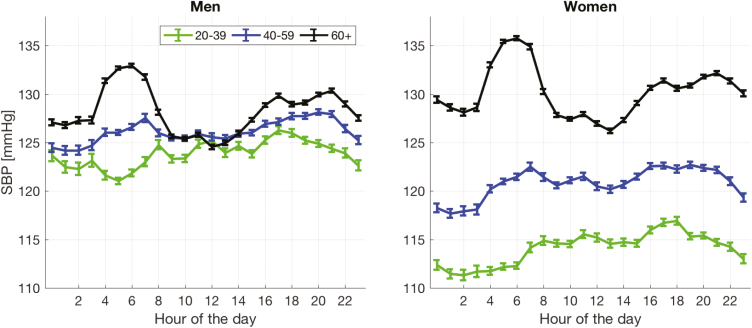
Twenty-four-hour (mean ± SE) systolic blood pressure for men (left) and women (right) for three age groups 18–39, 40–59 and 60+.

The 24-hour systolic BP variation for different age groups (Figures 3 and 4) shows a progressive change from the lowest recorded BP being at night in the younger age groups (dipping pattern) to the highest recorded BP being at night in the older age groups (rising pattern). The heart rate variation is shown in Figure 3; with all age groups, the heart rate decreases during the night, having its lowest value just before awakening.

### Twenty-four-hour BP variation during physiological instability

The effect of physiological instability on the 24-hour systolic BP variations for hospitalized patients (Supplementary Figure S4) was assessed by constructing the 24-hour BP profile after removing all observation sets, which contained a vital sign other than BP having a weight greater than 0.

## DISCUSSION

Little is known about BP characteristics and its circadian variation in the hospital setting. Using a large hospital database, comprised 1.7 million measurements collected between March 2014 and May 2018 in Oxford (UK), we have analyzed the 24-hour BP variation for men and women. The recording of BP in the hospital setting over a number of days and nights allowed us to compute 24-hour BP profiles. We excluded patients who stayed in hospital less than 24 hours, those with fewer than 3 measurements, and who did not have either daytime measurements or nighttime measurements.

In-hospital variations of mean systolic and mean diastolic BP with age were similar to those observed in the outpatient populations^20,21^—see also Supplementary Table S2 and Supplementary Figure S3. The 24-hour BP patterns in the overall population are also similar to those described in outpatient populations using ABPM.^6,22^

The nocturnal dipping pattern was not observed with in-hospital patients from late middle age onwards. Instead, it was replaced by a late nocturnal rise in BP above the age of 60, such that the peak nighttime systolic BP was greater than the peak daytime systolic BP, with the mean nighttime systolic BP also greater than the daytime systolic BP (Supplementary Table S3). The effect remained in patients with and without hypertension. To the best of our knowledge, these circadian patterns in BP in hospitalized patients have not been described. However, the patterns seem to agree with previous ABPM studies in the community.^6,11^

ABPM studies are normally synchronized to waking.^22^ Our analysis may also be serendipitously synchronized with waking, as the most common time to measure BP in the hospitals was between 5:00 am and 5:59 am. The second most common time to measure BP was between 8:00 pm and 8:59 pm, suggesting nighttime rest may also be somewhat synchronized. These external hospital-imposed rhythms may contribute to the alignment of the circadian rhythms of patients, allowing the 24-hour BP curves for subpopulations stratified by age to show very clear patterns.

The European Society of Hypertension Position Paper on ABPM states that patients with a nocturnal rising pattern have poor cardiovascular prognosis.^23^ Taylor *et al*.,^4^ in their systematic review and meta-analysis of multiple ABPM studies, also reported that rising BP at night compared with the daytime is associated with increased risk of cardiovascular events. Cuspidi *et al*.^24^ in their recent review highlighted that although the literature on the clinical and prognostic implications of nocturnal rising, or reverse dipping, is very limited, it is a powerful marker of poor cardiovascular prognosis, after adjusting for traditional risk factors. The concept of chronotherapy, administering antihypertensive drugs to lower BP over 24 hours, while maintaining nocturnal dipping^25^ may help ameliorate these risks.

Comparison with studies investigating changes in BP is hampered by differences in definition. Nocturnal hypertension is normally characterized by a simple threshold (mean systolic BP during the night greater than 120 mm Hg).^9^ Dipping or nondipping is assessed somewhat arbitrarily: if the mean nighttime BP is lower than the mean daytime BP by 10% or more, then the BP variation is said to follow a dipping pattern.^7^ The definition of the nighttime and daytime periods over which BP values are averaged to obtain the mean values varies extensively. For example, Taylor *et al*. report 13 different definitions of nighttime and daytime periods. We chose our ranges to be comparable to those used in most studies.^7^ The traditional assessment of dipping or nondipping tends to disguise the late nocturnal BP rise, which was the main characteristic of systolic BP data observed for in-hospital patients above the age of 60, as the lower BP found between midnight and 1:59 am balances the 4:00 am–5:59 am rise in the computation of mean nighttime BP.

Our study has important limitations. First, it is a retrospective study using measurements made on the wards by nursing staff as part of their regular observations of in-hospital patients. Patients who have longer hospital stays will therefore contribute more data to a hospital database of vital-sign observations. We compensated for this by generating one 24-hour BP profile per patient, regardless of their length of hospital stay. Second, the question of selection bias also arises. The patients whose BP is of most concern to the nursing staff will have their BP taken most frequently, possibly more often at night. However, patients may also get observations at night as a result of their admission time and the hospital protocol. As a result, BP was frequently recorded across the 24-hour period.

Several factors suggest that our findings may be robust to bias in sampling. In general, BP is measured more frequently in hospital to detect hypotension rather than the nocturnal hypertension described here for older patients. The late nocturnal BP rise for these patients is not accompanied by increased heart rates (see lowest plots in Figure 3), as would be expected if the presence of readings in the time period were disproportionately caused by being less well. The expected nocturnal dip in BP remains in younger patients. Removing observations taken when the patient was otherwise physiologically unstable had little effect on our findings (Supplementary Figure S4). Finally, the 24-hour BP patterns for elective and emergency patients also showed similar characteristics (Supplementary Figure S5).

It is not known whether the higher nighttime BP levels are a result of a nurse waking a patient up to fit a cuff around their arm, in comparison to it being inflated automatically as with ABPM. There may be a mechanistic link to the taking of vital-sign observations. Regular interruptions during the night may cause sleep deprivation, especially in elderly patients, which will lead to increased sympathetic activity.^26^ The renin–angiotensin–aldosterone system is normally activated in the early morning before arousal as a result of sympathetic neuronal activation leading to increased BP, and this may be the mechanism underlying the observed late nocturnal BP rise in the elderly in-hospital patient population.^27^ However, if the rise in nighttime BP was solely the result of being woken up, the phenomenon would affect all patients, regardless of their age. That is not the case, because the dipping pattern is clearly maintained below the age of 50 for men and below the age of 40 for women. In addition, the disturbance caused by the recording of the vital-sign observations would also be expected to increase the heart rate. Our results show that heart rate decreases at night for all age groups, and so it can be argued that the arousal at the time of the vital-sign recording is unlikely to be the direct cause of the nocturnal rise in BP in elderly patients.

Future work will explore the effects of different admission times and of different primary reasons for admission. The association with comorbidities also requires investigation. Outside hospital, increased nighttime systolic BP is known to be a significant risk factor for cardiovascular events.^6^ Understanding whether in-hospital patterns encode increased cardiovascular risk and/or persist on discharge are the next steps required to place these observations in context.

## SUPPLEMENTARY MATERIAL

Supplementary material is available at American Journal of Hypertension online.

hpz130_suppl_CircadianBPsupp_AJH_revised2

## FUNDING

The research described in this paper is supported by the NIHR Biomedical Research Centre, Oxford, and by an EPSRC grant (EP/N024966/1—Intelligent Wearable Sensors for Predictive Patient Monitoring).

## DISCLOSURE

A.M. is funded by an EPSRC grant (EP/N024966/1—Intelligent Wearable Sensors for Predictive Patient Monitoring) and undertakes consultancy work for Sensyne Health. P.W. works part-time for Sensyne Health and has share options in Sensyne Health. L.T. is a nonexecutive Director of Sensyne Health and holds share options in the company. R.J.M. has received BP monitors for research purposes from Omron and travel expenses and honoraria for speaking from the Japanese Society of Hypertension and the American Society of Nephrology.
