# Supplementary material for: Circadian Blood Pressure Variations Computed From 1.7 Million Measurements in an Acute Hospital Setting
Source: Am J Hypertens. 2019 Aug 16;32(12):1154–61. doi: 10.1093/ajh/hpz130 (PMC7427624; doi:10.1093/ajh/hpz130)
Supplement: hpz130_suppl_CircadianBPsupp_AJH_revised2 [file hpz130_suppl_circadianbpsupp_ajh_revised2.docx]

**Supplementary Material**

# Circadian blood pressure variations computed from 1.7 million measurements in an acute hospital setting

A. Mahdi^1^, P. Watkinson^2,4^, R.J. McManus^3^ and L. Tarassenko^1,4^

*^1^Institute of Biomedical Engineering, Department of Engineering Science, University of Oxford*

*^2^Nuffield Department of Clinical Neurosciences, Oxford University Hospitals NHS Trust, Oxford*

*^3^Nuffield Department of Primary Care Health Sciences, University of Oxford*

*^4^Sensyne Health, Schrödinger Building, Heatley Road, Oxford Science Park, Oxford, OX4 4GE*

### 24-hour BP signature

To compute the most representative BP for each hour of the day or night, for the kth patient, we first compute a 24-dimensional vector:

|  | $\mathbf{P}_{\mathbf{hour}}^{k}= \left. \left[ {\bar{P}^{k}}_{\mathbf{0}},{\bar{P}^{k}}_{\mathbf{1}},\ldots,{\bar{P}^{k}}_{\mathbf{23}} \right. \right]$ | (1) |
| --- | --- | --- |

where ${\bar{P}^{k}}_{j}$ is the average of all systolic (or diastolic) blood pressure values for the kth patient recorded between hour j and hour j+1 for j=0, …, 23. (j = 0 corresponds to the one-hour interval from midnight to 0:59 am; j = 23 to the one-hour interval from 11:00 pm to 11:59 pm). For most patients, some of the components of $\mathbf{P}_{\mathbf{hour}}^{k}$ will be empty (missing data) as these patients will not have had any measurements of BP taken for some of the 24 one-hour intervals. All patients each contribute one vector $\mathbf{P}_{\mathbf{hour}}^{k}$ of average values of systolic (or diastolic) blood pressure regardless of their length of stay in hospital and number of BP measurements recorded.

The 24-hour BP vector, for any group of patients, is computed as

|  | $\boldsymbol{P}_{\mathrm{hour}}=[\frac{1}{N_{0}}\sum_{k=1}^{N_{0}} {\bar{P}^{k}}_{0},\ldots, \frac{1}{N_{23}}\sum_{k=1}^{N_{23}} {\bar{P}^{k}}_{23}]$ | (2) |
| --- | --- | --- |

where *N*_0_ = number of average BP values for the interval from midnight to 0:59 am for that group of patients; similarly, *N*_23_ = number of average BP values for the interval from 11:00 pm to 11.59 pm.

Figures SF1 and SF2 illustrate the systolic BP data for two patients, one patient with 23 measurements (length of stay of 4.6 days) and one patient with 43 measurements (length of stay of 12.8 days). The BP values are displayed according to the time of the day or night at which they were recorded, with one-hour quantisation on the time axis.

The black crosses show the individual BP measurements taken on different days plotted against the relevant one-hour interval (the measurement being plotted at the *x*-coordinate corresponding to the left-hand edge of each interval). The BP value shown as a red dot is the average of the measurements shown as black crosses in that one-hour interval. The red dots are the individual components of the vector $\boldsymbol{P}_{\mathbf{hour}}^{k}$ from Equation (2) . When there is only one value for a particular one-hour interval, the red dots and black crosses coincide. Any one-hour interval for which there are no BP measurements is shown shaded in grey.

### Age variations of systolic and diastolic BP

Here we computed the mean systolic BP and the mean diastolic BP, for each patient, from all the available measurements for that patient during their hospital stay. Each patient only contributes one value, their mean systolic or (diastolic) BP over their entire hospital stay, per admission, regardless of their length of stay. We then averaged these data for all patients of the same sex in the eight age groups: 16-29, 30-39, 40-49, 50-59, 60-69, 70-79, 80-89 and 90+. Table ST1 and Figure SF3 show that in-hospital variations of systolic and diastolic BP with age were as expected from the literature.

The plots for any individual patient can show a high degree of variability. For example, in Figure SF2, the highest value of systolic BP (170 mmHg) occurs within the 10:00 am-10:59 am interval, and the lowest value occurs within the 12 noon-12:59 pm interval (60 mmHg).

| 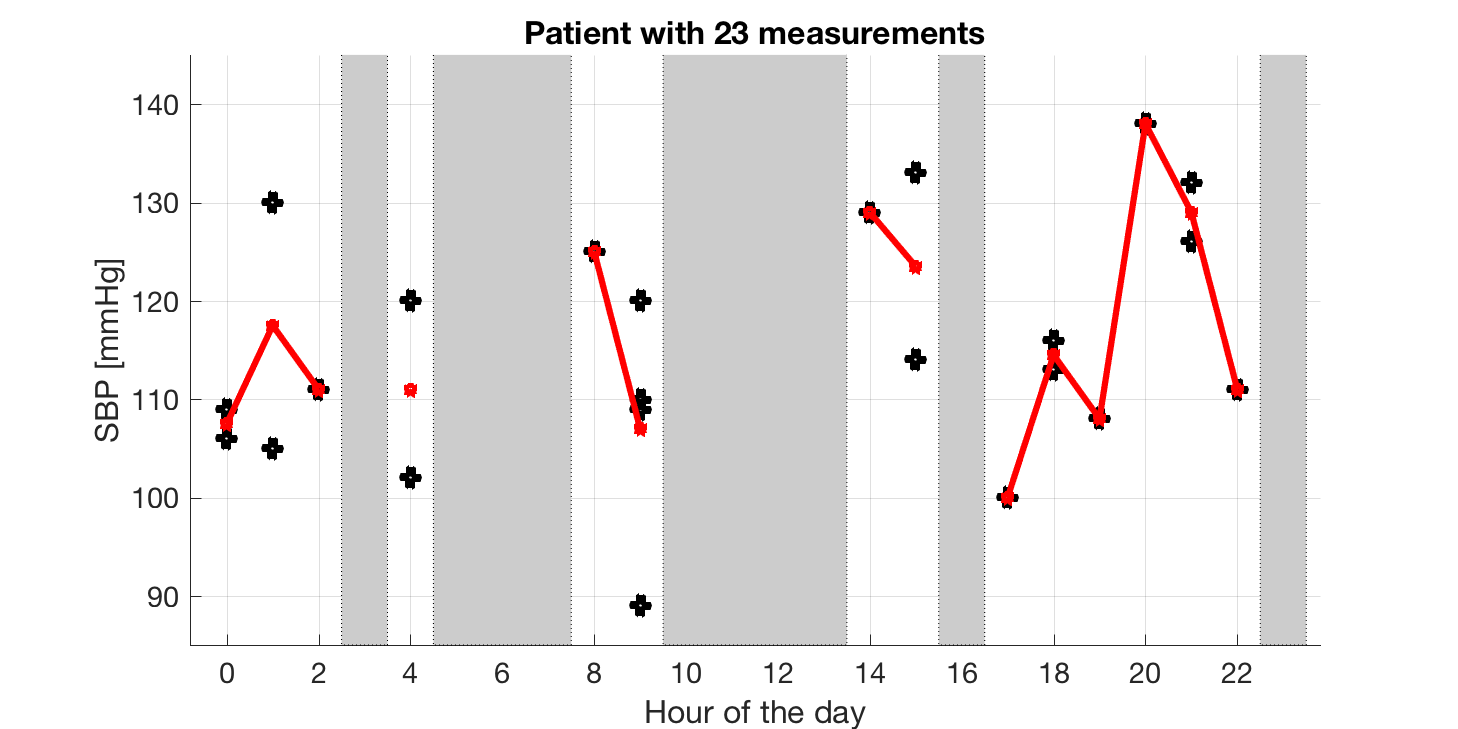  **Figure SF1**. 24-hour average plot of systolic blood pressure for a patient with 23 measurements |
| --- |


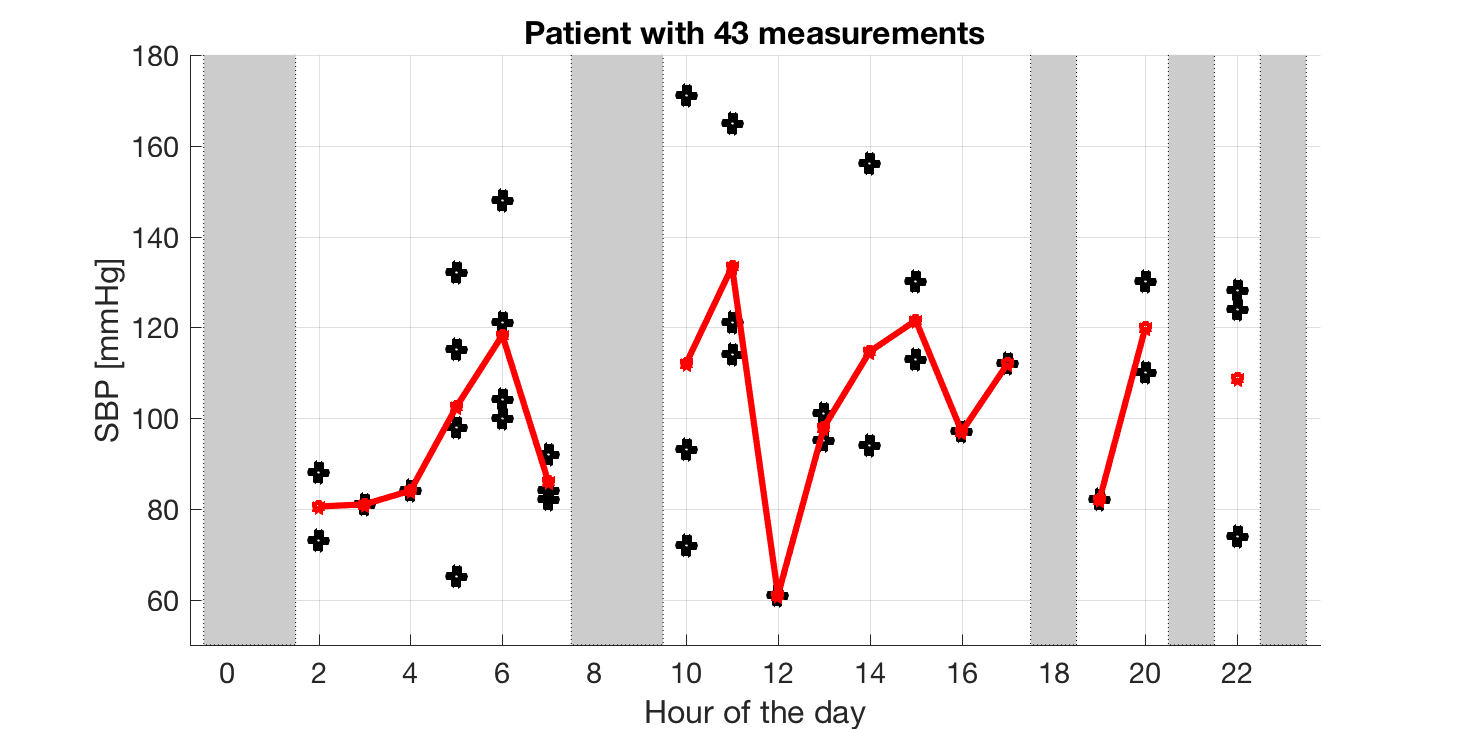


**Figure SF2**. 24-hour average plot of systolic blood pressure for a patient with 43 measurements

**Table ST1**. Demographic descriptors for included patients for each group of interest.

|  | **Patients** | | **Age** | **LOS** | **Obs** | **CCI** | **IHM** | **TA** |
| --- | --- | --- | --- | --- | --- | --- | --- | --- |
|  | N (%) | | Mn (SD) | Md (IQR) | Md (IQR) | Md (IQR) | N (%) | N (%) |
| All | 41,455 (100) | | 64 (19) | 4.7 (7.4) | 27 (34) | 3 (10) | 2,233 (5) | 20,480 (49) |
| Men | 20,169 (49) | | 64 (19) | 4.9 (7.5) | 28 (34) | 3 (10) | 1,175 (6) | 9,818 (49) |
| Women | 21,286 (51) | | 65 (20) | 4.6 (7.2) | 26 (33) | 0 (9) | 1,058 (5) | 10,662 (50) |
| **Age groups** |  | |  |  |  |  |  |  |
| 16-29 years | 3,019 (7) | | 23(4) | 2.9 (3.9) | 18 (19) | 0 (0) | 5 (0) | 1,965 (58) |
| 30-39 years | 2,722 (7) | | 35 (3) | 3.2 (4.5) | 19 (23) | 0 (0) | 19 (1) | 1,713 (63) |
| 40-49 years | 3,714 (9) | | 45 (3) | 3.6 (5.1) | 22 (26) | 0 (4) | 38 (1) | 2,299 (62) |
| 50-59 years | 5,674 (14) | | 55 (3) | 4.1 (5.8) | 25 (28) | 0 (4) | 113 (2) | 3,418 (60) |
| 60-69 years | 7,071 (17) | | 65 (3) | 4.5 (6.4) | 27 (32) | 3 (8) | 246 (3) | 4,084 (58) |
| 70-79 years | 8,680 (21) | | 74 (3) | 5.2 (7.9) | 30 (35) | 4 (12) | 503 (6) | 4,390 (51) |
| 80-89 years | 7,861 (19) | | 84 (3) | 6.8 (10.9) | 34 (44) | 8 (15) | 845 (11) | 2,367 (30) |
| 90+ years | 2,714 (7) | | 91 (1) | 7.5 (12.0) | 34 (45) | 11 (17) | 464 (17) | 453 (17) |
| **Hypertension category** | |  | | | |  |  |  |
| Normotensives (60+) | 8,016 (17) | | 74 (9) | 0.6 (3.1) | 6 (17) | 0 (8) | 1478 (3) | 13,968 (30) |
| Hypertensives (60+) | 18,310 (27) | | 77 (9) | 2.0 (6.2) | 13 (29) | 4 (14) | 3000 (4) | 19,870 (20) |

* LOS - length of stay (days); Obs – observations; CCI - Charlson Comorbidity Index; IMH - In hospital mortality; TA – hospital theatre admissions. N, Mn, Md, SD and IQR are the number, mean, median, standard deviation and inter-quartile range, respectively.

** Charlson Comorbidity Index and definitions of surgical specialties and elective admissions were determined according to the methodology and specification provided by NHS Digital (Charlson Comorbidity Index guidelines are available at <https://beta.digital.nhs.uk/publications/ci-hub/summary-hospital-level-mortality-indicator-shmi>).

**Table ST2**. Mean systolic (SBP) and diastolic (DBP) blood pressure (mmHg) for all patients,

men, women and eight age groups.

|  | **mean SBP** | | | **mean DBP** | | |
| --- | --- | --- | --- | --- | --- | --- |
|  | **All** | **Men** | **Women** | **All** | **Men** | **Women** |
| All | 127 | 127.5 | 127 | 69 | 70 | 68 |
| **Age groups** |  |  |  |  |  |  |
| 16-29 | 117 | 122 | 113 | 66 | 67 | 65 |
| 30-39 | 1190 | 124 | 115 | 69 | 72 | 67 |
| 40-49 | 122 | 125 | 119 | 71 | 74 | 68 |
| 50-59 | 125 | 127 | 124 | 71 | 74 | 69 |
| 60-69 | 127 | 128 | 127 | 70 | 72 | 67 |
| 70-79 | 130 | 129 | 131 | 68 | 69 | 67 |
| 80-89 | 132 | 130 | 134 | 68 | 68 | 68 |
| 90+ | 133 | 130 | 135 | 68 | 68 | 69 |

| 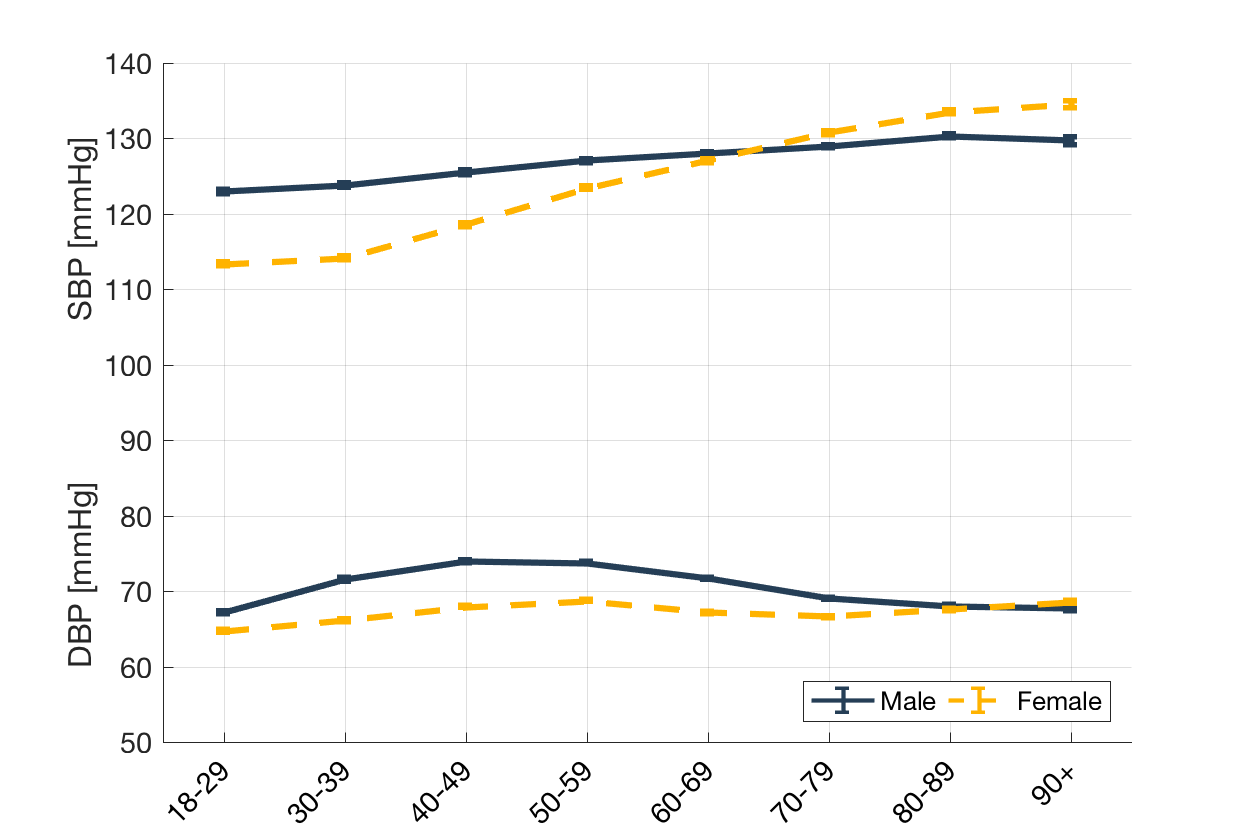  **Figure SF3**. Age-dependent m$ean\pm SE$ systolic and diastolic blood pressure for men and women, where SE is the standard error. |
| --- |

**Table ST3.** Mean nighttime (midnight - 5:59 am) and mean daytime (10:00 am - 7:59 pm) systolic blood pressure (mmHg) and the difference between the two values for different cohorts of patients.

|  | Men | | | Women | | |
| --- | --- | --- | --- | --- | --- | --- |
| Cohorts | Nighttime  mean SBP  (95% CI) | Daytime  mean SBP  (95% CI) | Difference  (95% CI) | Nighttime  mean SBP  (95% CI) | Daytime  mean SBP  (95% CI) | Difference  (95% CI) |
| All | 127.9 (0.3) | 127.0 (0.2) | -0.9 (0.4) | 126.7 (0.3) | 125.8 (0.2) | -0.9 (0.3) |
| Hypertensives (60+) | 132.2 (0.4) | 128.8 (0.3) | -3.4 (0.5) | 135.5 (0.4) | 131.4 (0.4) | -4.1 (0.5) |
| Normotensives (60+) | 125.8 (0.5) | 125.2 (0.5) | 0.5^*^ (0.7) | 126.2 (0.5) | 125.5 (0.5) | -0.7^**^ (0.7) |
| 20-39 years | 121.4 (0.6) | 125.6 (0.5) | 3.2 (0.7) | 111.8 (0.5) | 115.2 (0.5) | 3.4 (0.7) |
| 40-59 years | 125.2 (0.5) | 126.7 (0.4) | 1.5 (0.7) | 119.5 (0.5) | 121.9 (0.4) | 2.4 (0.7) |
| 60+ years | 130.4 (0.3) | 127.8 (0.3) | -2.6 (0.4) | 132.5 (0.3) | 129.5 (0.3) | -3.0 (0.4) |

CI – confidence intervals; Difference – difference between the corresponding nighttime mean SBP (mmHg) and daytime mean SBP (mmHg). All differences are significant with P<0.001 except ^*^P<0.15 and ^**^P<0.05.


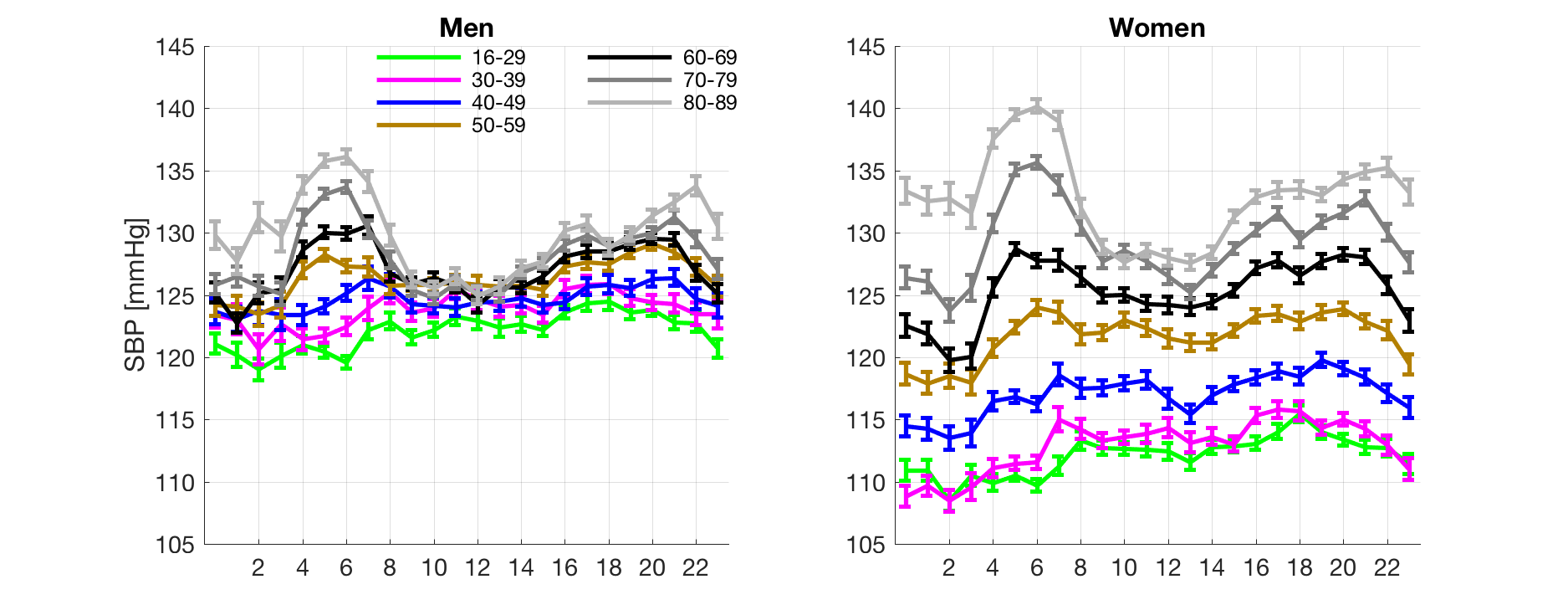


**Figure SF4**. 24-hour (m$ean\pm SE$) systolic blood pressure curves for different age groups for men and women with measurements corresponding to a weight > 0 for all vital signs except systolic blood pressure taken out. Here SE is the standard error.

**Figure SF5**. 24-hour (m$ean\pm SE$) systolic blood pressure curves for elective and emergency admission patients, where SE is the standard error.


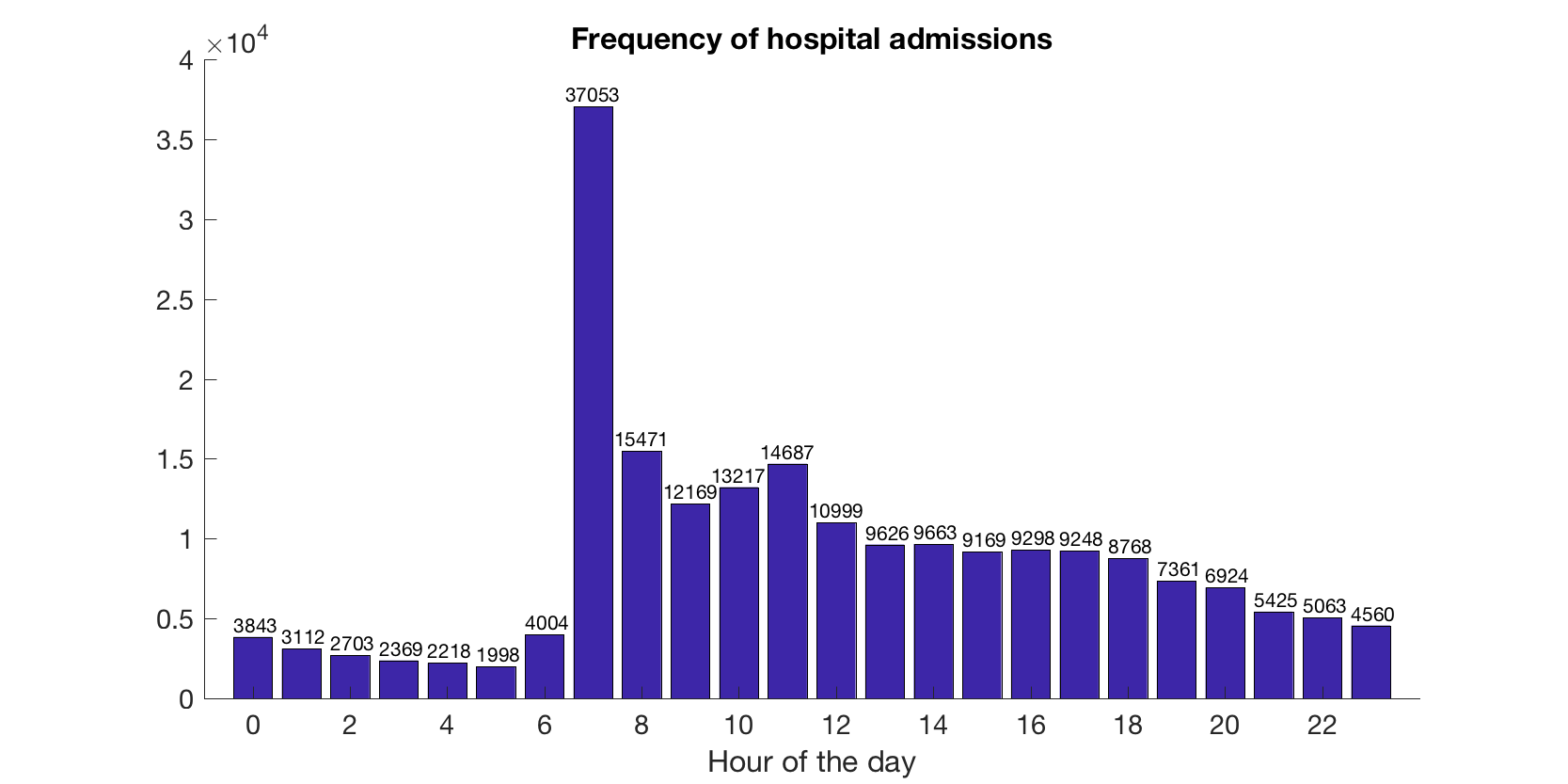


**Figure SF6.** The frequency of admission by hour of the day.

**Table ST4**. Anti-hypertensive drugs listed in the British National Formulary, March 2017

| **Beta blockers** | **Renin-angiotensin ACE inhibit** | **Renin angiotensin angio2receptor antagonist** |
| --- | --- | --- |
| Carvedilol | Captopril | Azilsartan medoxomil |
| Labetalol hydrochloride | Co-zidocapt | Candesartan cilexetil |
| Nadolol | Enalapril maleate | Eprosartan |
| Oxprenolol hydrochloride | Enalapril with hydrochlorothiazide | Irbesartan |
| Pindolol | Fosinopril sodium | Irbesartan with hydrochlorothiazide |
| Propranolol hydrochloride | Imidapril hydrochloride | Losartan potassium |
| Timolol maleate | Lisinopril | Losartan with hydrochlorothiazide |
| Acebutolol | Lisinopril with hydrochlorothiazide | Olmesartan medoxomil |
| Atenolol | Moexipril hydrochloride | Olmesartan with amlodipine |
| Bisoprolol | Perindopril arginine | Telmisartan |
| Celiprolol hydrochloride | Perindopril arginine with indapamide | Telmisartan with hydrochlorothiazide |
| Co-tenidone | Perindopril erbumine | Valsartan with hydrochlorothiazide |
| Esmolol hydrochloride | Quinapril |  |
| Metoprolol tartrate | Quinapril with hydrochlorothiazide |  |
| Nebivolol | Ramipril with felodipine |  |
|  | Trandolapril |  |

| **Calcium-channel blockers** | **Diuretics** |
| --- | --- |
| Amlodipine | Amiloride with cyclopenthiazide |
| Diltiazem hydrochloride | Bendroflumethiazide |
| Felodipine | Co-amilozide |
| Isradipine | Hydrochlorothiazide |
| Lacidipine | Indapamide |
| Lercanidipine hydrochloride |  |
| Nicardipine nydrochloride |  |
| Nifedipine |  |
| Verapamil hydrochloride |  |

| **Vasodilators Antihypertensives** | **Vasodilators Peripheral** | **Central Acting** |
| --- | --- | --- |
| Hydralazine hydrochloride | phenoxybenzamine hydrochloride | Clonidine hydrochloride |
| Minoxidil | phentolamine mesilate | Methyldopa |
| Sodium nitroprusside |  | Moxonidine |

| **Renin angiotensin renin inhibit** | **Antiadrenergic** |  |
| --- | --- | --- |
| Aliskiren | Guanethidine monosulfate |  |

**Table ST5**. ICD-10 codes for hypertension

| **I10: Essential (primary) hypertension** |
| --- |

| **I11: Hypertensive heart disease** | |
| --- | --- |
| **I11.0** | Hypertensive heart disease with (congestive) heart failure |
| **I11.9** | Hypertensive heart disease without (congestive) heart failure |

| **I12: Hypertensive renal disease** | |
| --- | --- |
| **I12.0** | Hypertensive renal disease with renal failure |
| **I12.9** | Hypertensive renal disease without renal failure |

| **I13: Hypertensive heart and renal disease** | |
| --- | --- |
| **I13.0** | Hypertensive heart and renal disease with (congestive) heart failure |
| **I13.1** | Hypertensive heart and renal disease with renal failure |
| **I13.2** | Hypertensive heart and renal disease with both (congestive) heart failure and renal failure. |
| **I13.9** | Hypertensive heart and renal disease, unspecified |

| **I15: Secondary hypertension** | |
| --- | --- |
| **I15.0** | Renovascular hypertension |
| **I15.1** | Hypertension secondary to other renal disorders |
| **I15.2** | Hypertension secondary to endocrine disorders |
| **I15.8** | Other secondary hypertension |
| **I15.9** | Secondary hypertension, unspecified |
